# Comparison of CT and PET-CT based planning of radiation therapy in locally advanced pancreatic carcinoma

**DOI:** 10.1186/1756-9966-27-41

**Published:** 2008-09-23

**Authors:** Erkan Topkan, Ali A Yavuz, Mehmet Aydin, Cem Onal, Fuat Yapar, Melek N Yavuz

**Affiliations:** 1 Department of Radiation Oncology, Baskent University Medical Faculty, Adana Medical and Research Center, Kisla Saglik Yerleskesi, 01120, Adana, Turkey; 2 Department of Nuclear Medicine, Baskent University Medical Faculty, Adana Medical and Research Center, Kisla Saglik Yerleskesi, 01120, Adana, Turkey

## Abstract

**Background:**

To compare computed tomography (CT) with co-registered positron emission tomography-computed tomography (PET-CT) as the basis for delineating gross tumor volume (GTV) in unresectable, locally advanced pancreatic carcinoma (LAPC).

**Methods:**

Fourteen patients with unresectable LAPC had both CT and PET images acquired. For each patient, two three-dimensional conformal plans were made using the CT and PET-CT fusion data sets. We analyzed differences in treatment plans and doses of radiation to primary tumors and critical organs.

**Results:**

Changes in GTV delineation were necessary in 5 patients based on PET-CT information. In these patients, the average increase in GTV was 29.7%, due to the incorporation of additional lymph node metastases and extension of the primary tumor beyond that defined by CT. For all patients, the GTV_CT _versus GTV_PET-CT _was 92.5 ± 32.3 cm^3 ^versus 104.5 ± 32.6 cm^3 ^(*p *= 0.009). Toxicity analysis revealed no clinically significant differences between two plans with regard to doses to critical organs.

**Conclusion:**

Co-registration of PET and CT information in unresectable LAPC may improve the delineation of GTV and theoretically reduce the likelihood of geographic misses.

## Background

Surgery offers the only potential cure for pancreatic carcinoma; however, more than 85% of patients have tumors that are not amenable to surgical resection, due to advanced disease at presentation [1,2]. Although 45% of those patients present with metastatic disease, the remaining 40% present with unresectable, locally advanced pancreatic carcinoma (LAPC). These patients still have a theoretical chance for cure with non-surgical treatment, such as systemic chemotherapy and/or radiation therapy (RT) [2,3]. However, in unresectable LAPC, reported local relapse rates after RT are high, ranging from 42% to 68% [4,5]; these rates may be associated with either geographic misses or the insufficiency of conventional radiation doses of 45–50 Gy.

An obvious way to reduce geographic misses in pancreatic carcinoma is through a more accurate definition of RT target volumes. However, contrast-enhanced computerized tomography (CT), which is the current standard imaging modality for planning three-dimensional conformal RT (3D-CRT), has a relatively low sensitivity and specificity in determining the extent of primary tumor and nodal involvement [6-13], which directly impact the definition of target volumes. Therefore, it is desirable to supplement CT when defining gross tumor volume (GTV) and its subsequent expansion to planning target volume (PTV).

In several tumor sites including pancreas, functional 18F-fluoro-deoxyglucose positron emission tomography (FDG-PET) has been shown to have higher sensitivity and specificity than anatomic CT in the detection of primary tumors, lymphatic extensions, and distant metastases [14-18]. Potential roles of FDG-PET in imaging pancreatic carcinoma have been demonstrated [8-10,12,19], but the precise delineation of positive findings is hampered by limited anatomic information provided by PET images [20,21]. However, this limitation may be overcome by using co-registered functional PET and anatomic CT images, which may further improve delineation of target volumes by providing better discrimination between malignant and benign lesions and determining lymphatic status. PET-CT based RT planning (RTP) has been shown to significantly alter RT fields in patients with various tumors, such as cancers of the head and neck, lung, and esophagus [22-24]. However, to our knowledge, there is no report on the role of PET-CT based RTP in patients with unresectable LAPC. We hypothesized that using PET-CT data rather than CT data alone would change RT fields and possibly result in fewer geographic misses for pancreatic region. In this current study, we compared CT- and PET-CT-based GTV delineation and its subsequent expansion to the PTV; we also analyzed the resultant doses of 3D-CRT to critical organs.

## Methods

### Patient population

Fourteen patients with pathologically confirmed, unresectable LAPC were prospectively enrolled. Other eligibility criteria were as follows: Eastern Cooperative Oncology Group performance status (PS) of 0 to 2; age between 18 and 70 years; determination of disease extent by laparotomy or laparoscopy, and radiographic imaging; no prior chemotherapy or abdominal irradiation; no contraindication for PET-CT imaging. All patients provided written informed consent, and the study design was approved by the institutional ethics committee, in accordance with Helsinki Declaration on human projects.

### CT and PET imaging

Prior to the CT and PET imaging, patients were immobilized in customized alpha cradles in the simulation position, which is supine with arms up. Before imaging, simulator lasers (Acuity, Varian Medical Systems, Palo Alto, CA, USA) were used to align and mark patients to define the coordinate system that would be used for treatment planning.

For PET-based planning, eligible patients were investigated with the combined PET-CT system (Discovery-STE 8, General Electric Medical System, Milwaukee, WI, USA). The patients fasted for at least 6 hours before administration of intravenous 370–555 MBq (10–15 mCi) FDG. Pre-injection blood glucose levels were measured to ensure that they were below 150 mg/dl. During the distribution phase the patients lay supine in a quiet room. The combined image acquisition started 60 minutes after the FDG injection. The patients were scanned on the flat-panel carbon fiber composite table insert. At first, an unenhanced CT scan (5 mm slice thickness) from the base of the skull to the inferior border of the pelvis was acquired, using a standardized protocol with 140 kV, 80 mA. The subsequent PET scan was acquired in 3D mode from the base of the skull to the inferior border of the pelvis (6–7 bed positions, 3 min per bed position) without repositioning the patient on the table. Both CT and PET images were acquired with the patient breathing shallowly. Attenuation was corrected by using the CT images. Areas of FDG uptake were categorized as malignant based on location, intensity, shape, and size and visual correlation with CT images to differentiate physiologic from pathologic uptake.

### Image registration and radiation treatment planning

Eclipse 7.5 (Varian Medical Systems, Palo Alto, CA, USA) RTP system, which includes all standard RTP features as well as DICOM image read and the automated image registration software, was used to perform CT- and PET-CT-based treatment planning for each patient. Two radiation oncologist contoured the GTV, the PTV, and the normal organs of interest on the CT images, independent of the PET images. For purposes of this study, a nuclear medicine physician outlined the lesion on the registered PET images. We set the window and level for the PET images according to the method previously described by Erdi et al. [25] for accurate target volume definition. In this protocol, we first measured the value of the hottest pixel in the lesion and then set the upper window level to this maximum value and lower window level to 42% of the maximum level.

The target volumes were consensually defined by two radiation oncologists with specific experience in pancreatic cancer treatment. Despite of their large experience on delineating target volumes in this disease site, we addressed the possible interobserver variability between two observers in 6 sampled patients. The variability was less than 0.4 mm for GTV and 0.5 mm for PTV delineations, respectively. For each patient, the GTV_CT _was contoured to include the visible primary tumor and involved peripancreatic lymph nodes (≥1.5 cm in short axis). On the other hand, GTV_PET-CT _contouring included the primary tumor and the lymph nodes that appeared to be involved on either of CT (≥1.5 cm in short axis) and/or PET images irrespective of their size. For each method, our PTV was defined as GTV + 2 cm in each direction, to allow for microscopic extension and patient motion. To facilitate the comparison of methods, the three dimensional treatment plans and dose volume histograms (DVHs) for CT- only and PET-CT- based data were generated separately for each patient.

We planned to treat a single target volume with no cone down volumes. Treatment volumes were defined by using customized multileaf collimation. A four-field technique (posteroanterior, anteroposterior, and laterals) was mandated. A dose of 50.4 Gy (1.8 Gy/fr) was prescribed to encompass the defined PTV with isodose lines not 'cooler' than 95% and not 'hotter' than 107%. To achieve this, we used dosimetric practice wedges to modify beams. The source to axis distance was 100 cm. For each method, RTPs and DVHs were generated for 18 MV photon energy. For normal tissues, the maximum dose limits were of 45 Gy for the spinal cord, 50 Gy for the small bowel and stomach, 36 Gy for the liver, and 20 Gy to at least two thirds of one functioning kidney.

### Statistical analysis

On the basis of the literature concerning other tumor types, we hypothesized that integration of PET into RTP would change the target volumes in approximately 30% of the patients. In order to detect such a change with a 95% confidence interval of 5–55%, we needed to enroll at least 13 patients.

Statistical differences between paired parameters from CT- versus PET-CT-based treatment plans were evaluated with the Wilcoxon signed rank test. Results are either expressed as mean ± Standard deviation (SD) or as a proportion of 95% confidence intervals (95% CI). Differences were considered significant when the two-tailed *p*-value was less than 0.05.

## Results

**Figure 1:**
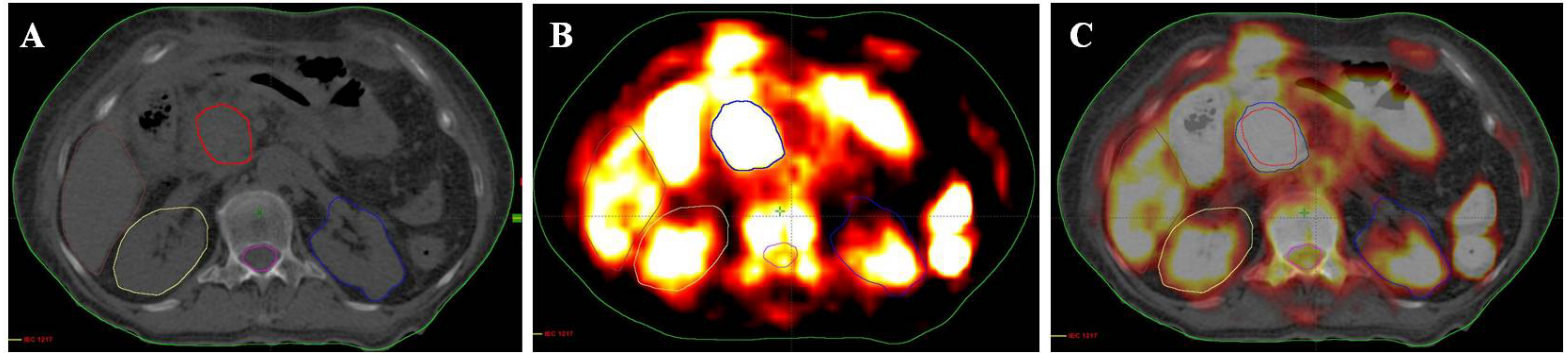
Representative image of a patient with different GTV delineations; CT (A), PET (B), and co-registered PET-CT.

The pretreatment tumor and patient characteristics for the 14 analyzable patients are summarized in Table 1. Although there was a need to change in both GTV and PTV based on PET information in all of the patients, this need was more prominent in 5 (35.7%) of them. The mean increase in GTV and corresponding PTV were; 29.7% (95% CI: 18.2–40.6) and 13.4% (95% CI: 8.6–21.3,) in this patients group (Figure 1). The reasons for these changes in GTV were detection of primary tumor beyond the CT-defined tumor boundaries in 4 (28.5%) patients, and detection of additional lymph node metastases in 1 (7.2%). The mean increase in GTV was 29.7% (95% CI: 18.2–40.6) in this patients group. Compared to CT-based delineation, the PET-CT-based delineation resulted in a significant increase in both the mean GTVs and the mean PTVs in the whole study population (Table 2).

**Table 1:** Pretreatment tumor and patient characteristics

Patient	Gender	Age (year)	Performance (ECOG)	Pancreatic Primary	Clinical TN-stage	SUV_max_
1	Male	63	2	Head	T_4_N_0_	9.8
2	Male	61	1	Head	T_4_N_1_	10.9
3	Male	54	1	Head	T_4_N_1_	11.3
4	Female	65	1	Body	T_4_N_1_	19.2
5	Male	48	0	Head	T_4_N_0_	14.7
6	Male	52	0	Body	T_4_N_1_	8.8
7	Female	59	2	Head	T_4_N_0_	7.9
8	Male	57	1	Head	T_4_N_1_	10.4
9	Male	46	1	Head	T_4_N_1_	17.6
10	Male	51	0	Head	T_4_N_0_	15.3
11	Male	58	0	Body	T_4_N_1_	8.6
12	Female	62	1	Head	T_4_N_1_	9.3
13	Male	43	1	Body	T_4_N_0_	11.5
14	Male	55	2	Head	T_4_N_1_	15.8

*Abbreviations: *ECOG = Eastern Cooperative Oncology Group; SUV_max _= Maximum standard uptake value

**Table 2:** Treatment results

Parameters	CT	PET-CT	P
GTV (cm^3^)			
Mean ± S.D.	92.5 ± 32.3	104.5 ± 32.6	0.009
Min-max	50.0–161.2	57.8–167.4	
			
PTV (cm^3^)			
Mean ± S.D.	479.4 ± 103.4	535.2 ± 96.2	0.008
Min-max	337.4–708.4	376.5–728.2	
			
Spinal cord dose (%)			
Mean ± S.D.	22.8 ± 5.7	23.0 ± 5.3	0.22
Min-max	13.1–31.6	15.9–32.2	
			
Liver dose (%)			
Mean ± S.D.	36.4 ± 7.3	37.3 ± 7.5	0.17
Min-max	26.1–50,1	27.1–50.8	
			
Stomach dose (%)			
Mean ± S.D.	42.3 ± 11. 7	46.3 ± 14.5	0.08
Min-max	19.7–59.1	24.4–66.8	
			
Left kidney dose (%)			
Mean ± S.D.	23.0 ± 5.0	23.9 ± 5.1	0.12
Min-max	14.2–33.5	15.9–31.3	
			
Right kidney dose (%)			
Mean ± S.D.	44.9 ± 8.7	50.2 ± 11.2	0.03
Min-max	30.8–62.8	28.7–66.2	

*Abbreviations: *PTV = Planning target volume; GTV = Gross tumor volume; S.D = Standard deviation

An overview of the radiation exposure of normal tissues, comparing CT- and PET-based plans, is shown in Table 2. Based on the DVHs for the two RTPs, there was no clinically significant difference in the percentage of the PTV dose that was received by critical organs other than the right kidney. However, for the right kidney, the PET-based delineation of the target volumes resulted in a mean increase in radiation exposure by 5.3%.

## Discussion

We compared CT- and PET-CT-based target volume delineation for unresectable LAPC and the effects of these different modalities on 3-D-CRT planning and radiation doses to critical organs. Our results demonstrated that PET-CT-based target volume contouring significantly increases the GTV and the PTV compared to CT-based contouring without increasing tissue toxicity in a clinically meaningful way.

The current treatment of choice for patients with unresectable LAPC consists of concurrent or sequential chemo-radiotherapy. In spite of significant improvements in chemotherapy and radiation oncology, the observed rates of local control remain far from acceptable limits. A theoretically reasonable way to reduce local recurrence is the accurate definition of target volumes and administration of appropriate radiation doses to these volumes. However, the accurate definition and contouring of the boundaries of the primary tumor and its locoregional extension are difficult with conventional imaging modalities, and a significant inter-observer variation has been demonstrated. Compared to non-registered images the co-registration of CT and PET images reduces inter-observer variability for different tumor types [26-28], and offers a higher sensitivity and accuracy in delineating primary tumors and lymph node metastases [14,29]. In a series of 52 patients with pancreatic carcinoma, Delbeke et al. [30] demonstrated that FDG-PET had a higher sensitivity, specificity and accuracy than CT in diagnosing pancreatic carcinoma (92%, 85% and 91% for FDG-PET versus 65%, 61% and 65% for CT, respectively). In another study, Lemke et al (31) compared the diagnostic value of CT, PET, and PET-CT fusion in 104 patients with susceptible pancreatic lesions. The authors reported that the image fusion improved the sensitivity of malignancy detection from 76.6% (CT) and 84.4% (PET) to 89.1% (image fusion). Compared to CT alone, image fusion increased the sensitivity of detecting infiltration of adjacent tissue from 47.7% to 68.2%. Based on the above literature, we hypothesized that the accuracy of target volume delineation might be improved beyond the CT-based delineation by integrating PET and CT data. This integration provides anatomical and functional information that may positively alter the treatment of patients with LAPC undergoing 3D-CRT.

In RTP studies of various tumors, both an increase and decrease in GTV have been observed by adding PET information to other imaging data [22-24]. While this research may form the basis for using PET imaging for RTP in pancreatic cancers, to our knowledge, no studies have been published specifically on the impact of PET imaging on the treatment of LAPC. In head and neck [6-33], lung [23,29] and gynecologic carcinomas [32,34] the increase and decrease in GTV associated with adding PET information have been demonstrated. The increase in GTV was attributed to the detection of primary tumor beyond the CT defined tumor boundaries, the detection of additional lymph node metastases, or both; on the other hand, the reduction in GTV was almost always associated with the exclusion of nodal metastases. Limited information is available concerning the role of PET in RTP for tumors of the alimentary tract. Vrieze et al. [35] reported the increase in GTV in 3 of 30 esophageal carcinomas, based on the detection of additional pathologic lymph nodes using FDG-PET rather than CT. Leong et al. [24] reported the comparison of GTV_CT _and GTV_PET _in 10 esophageal carcinomas. In 90% of patients, PET positive lesions were located outside GTV_CT _and in 30% outside the CT-based PTV. Ciernik et al. [32] observed an increase in GTV in 3 of 6 (50%) of patients with rectal carcinoma, and this increase in GTV lead to a 20% increase in PTV. In another report, Lammering et al. [36] studied a group of 40 patients with rectal carcinoma and demonstrated a significant increase in GTV with PET information (GTV_CT _95.9 ± 57.1 cm^3 ^versus GTV_PET _128.3 ± 80.4 cm^3^; *P *< 0.001). Although we studied a different tumor site (pancreas), similar with other alimentary tract primaries as mentioned above, our current results demonstrated that co-registered PET-CT findings resulted in an enlargement of the GTV outline in 5 of 14 (35.7%) patients with an average increase of 29.7% in delineated volume.

Given the overall poor outcome in patients with pancreatic cancer, one may intuitively question the efficacy of prophylactic irradiation of nodal groups as a standard component of disease management. However, to our best knowledge, this issue has never been addressed in a formal or prospective fashion. The M.D. Anderson Cancer Center (MDACC) has published the results of a phase 1 study using dose escalation of gemcitabine (350 mg/m^2 ^to 500 mg/m^2 ^weekly for 7 weekks) and hypofractionated RT (30 Gy in 10 fractions [24,37]. Grade 3–4 hematological and nonhematological toxicities were significant in all three arms, probably as a result of the larger RT fields. As an alternative strategy, Mc Ginn et al used reduced RT fields (PTV = GTV + 1 cm without elective nodal irradiation) in escalated doses of RT concurrent with gemcitabine in 34 unresectable or incompletely resected pancreatic carcinoma patients [38]. At the final planned dose level of study (42 Gy in 15 fractions) dose limiting toxicity was noted in two of six assessable patients. The authors concluded that the use of reduced RT fields did not affect the patterns of failure and the use of very conformal three-dimensional treatment planning may have positively altered the incidence of gastrointestinal toxicity. The PTV definition utilized by Mc Ginn and associates is obviously smaller than the one utilized in our current cohort. Based on the above favorable data we planned to not irradiate the CT and PET negative regional lymph nodes to an effort to decrease the possible gastrointestinal toxicity. However, when compared we have greater chance for incidental irradiation of microscopically involved lymph nodes. Furthermore, as we utilized PET data we have also included the regional lymph nodes which appeared to be metabolically involved on PET scan but not on CT. However, as the current study is going on, we think that it is more appropriate to wait for its growing clinical reflections to achieve more precise conclusions.

The present results are important for two reasons. First, in unresectable LAPC, the reported 42% to 68% local recurrence rate following RT even with chemotherapy is extremely high [4,5]. Excluding the ineffectiveness of current chemotherapeutics at this disease site, this recurrence rate may partly be due to geographic misses or the insufficiency of conventional 45–50 Gy doses. The need for an enlargement of the radiation field because of increased GTV based on PET information in 35.7% of our patients lends support for the possibility of geographic misses when CT information is used as the sole imaging tool in RTP. Second, regarding the doses to critical organs, the analysis of the CT and PET-CT-based DVHs revealed no clinically significant differences between the two RTPs in our study. This finding suggests the possibility of escalating the dose safely beyond the conventional doses, which may improve current rates of local control. However, such dose escalation would require further research in a similarly designed study, which is being tested in our ongoing study.

## Conclusion

This study demonstrated the usefulness of PET-CT based target volume delineation in patients with LAPC. The largest potential benefit of incorporating PET into RTP for unresectable LAPC may be the reduction in geographic misses associated with CT-based planning, and, as a result, the potential reduction in local and regional treatment failures. However, we believe that, before reaching more precise conclusions more clinical studies are needed to better define the role of PET-CT fusion in this setting.

## Competing interests

The authors declare that they have no competing interests.

## Authors' contributions

ET carried out all CT evaluations, study design, delineation of target volumes, statistical analysis, interpretation of the study, and drafted the manuscript. AAY participated in study design and interpretation of the study. MA and FY carried out all PET evaluations and delineation of target volumes based on PET findings. CO participated in manuscript preparation and study design. MNY participated in manuscript preparation and study design. All authors read and approved the final draft.
